# Supplementary material for: Stereoelectronic and hydrogen-bonding effects on hydroxyproline conformation
Source: Commun Chem. 2026 Mar 24;9:179. doi: 10.1038/s42004-026-01984-x (PMC13184006; doi:10.1038/s42004-026-01984-x)
Supplement: Supplementary file 2 — Supplementray Material [file 42004_2026_1984_MOESM2_ESM.pdf]

Supplementary Information for: Stereoelectronic and Hydrogen-Bonding Effects on Hydroxyproline Conformation

Fumiki Matsumura[1], Pablo Gómez Argudo[1,3], Mischa Bonn[1], Giulia Giubertoni[2*], Johannes Hunger[1*]

[1]Department of the Molecular Spectroscopy , Max Planck Institute for Polymer Research, Ackermannweg 10, Mainz, 55128, Germany.
[2]Van 't Hoff Institute for Molecular Sciences, Science Park 904, Amsterdam, 1098XH, The Netherlands.
[3]Department of Physical Chemistry and Applied ThermodynamicsUniversity of Córdoba, Córdoba, 14014, Spain.

*Corresponding author(s). E-mail(s): g.giubertoni@uva.nl; hunger@mpip-mainz.mpg.de;

Supplemental Note 1: The effect of the explicit solvent on the DFT-calculated anisotropy

To estimate the effect of the interaction with the solvents on molecular conformation and the inferred anisotropy value, we tested the effect of explicit solvents. In addition to the implicit solvent DFT calculations presented in the main manuscript, we employed explicit solvents for the DFT calculations. Using the ORCA solvator, eight chloroform molecules were placed around the solute molecule, followed by geometry optimization with the M06-2X functional and cc-pVTZ basis set. We compared the anisotropy values obtained using the implicit conductor-like polarizable continuum model (CPCM, see main manuscript) with those obtained using CPCM combined with eight explicit chloroform molecules, as summarized in Supplementary Table 1.

As a result of implicit solvent molecules, anisotropy values can vary by up to ≈0.1. Therefore, we estimate the uncertainty of the DFT-calculated anisotropy to ≈0.1.

Supplementary Table 1 The anisotropy values of conformers (A)-(H) calculated with the implicit conductor-like polarizable continuum model (CPCM) and CPCM combined with eight explicit chloroform molecules.

Conformer	Anisotropy with CPCM	Anisotropy with CPCM and eight explicit solvents
A	-0.178	-0.197
B	0.167	0.322
C	-0.116	-0.175
D	0.255	0.246
E	-0.185	-0.154
F	0.123	0.133
G	-0.00440	-0.159
H	0.242	0.197

Supplemental Note 2: The absorption coefficient and transition dipole moment of the amide vibration

To estimate the amplitudes of the amide vibrations, we calculated the absorption coefficients from the DFT calculation as summarized in Table 2. We note that the absorption coefficient shown here is directly proportional to the square of the transition dipole moment[1].

Supplementary Table 2 The absorption coefficient of amide vibrations of conformers (A)-(H) from the DFT calculation.

Conformer	Absorption coefficient (L/mol*cm)
A	0.153
B	0.140
C	0.151
D	0.189
E	0.151
F	0.142
G	0.148
H	0.187

Supplemental Note 3: Peak fitting to determine peak amplitudes of amide and ester peaks

To estimate the relative abundance of the cis and the trans conformers of Boc-4R-Hyp-OMe and Boc-4S-Hyp-OMe in solution, we fitted five peaks to the infrared absorption spectra to model the three amide peaks and the two ester peaks, as identified in the main manuscript. The relative population of the cis and the trans conformer should be proportional to the amplitude ratio of the two corresponding amide peaks. However, these ratios are sensitive to the exact details of the fitted line shapes and line widths. To reduce the number of adjustable parameters, we constrain the peak maxima to the location of the minima in the second derivative spectra. We used Gaussian line shapes (Supplementary Figures 1 & 3) or Voigt bands (Supplementary Figures 2 & 4). To further reduce the number of adjustable parameters, we assume the linewidths of the amide peaks and the ester peaks to be the same for each spectrum separately (Supplementary Figures 1 & 2), or for both spectra of both diastereomers (Supplementary Figures 3 & 4). The thus determined ratios of the two amide peak amplitudes using these different assumptions are indicated in the figures and serve as an estimate for the population of the two conformations.

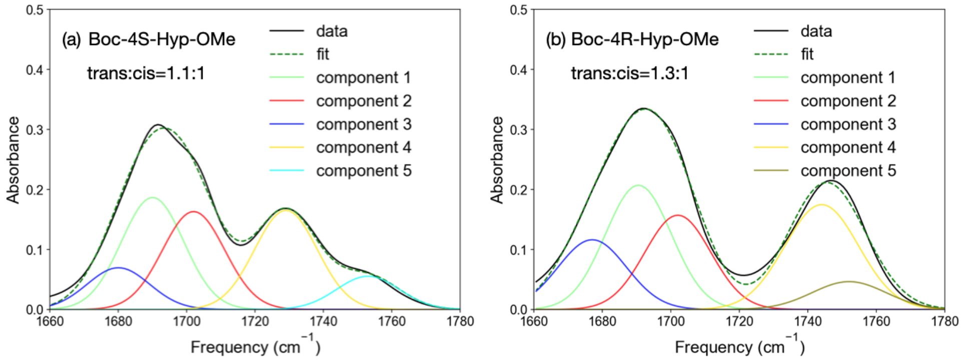

Supplementary Figure 1 The result of the peak fitting of (a)Boc-4R- and (b)Boc-4S-Hyp-OMe at 100 mM concentration using Gaussian line shapes. Each spectrum of Boc-4R- and Boc-4S-Hyp-OMe was fitted individually using Gaussian lineshapes. The peak positions were fixed based on the location of the minima in the second derivative spectra. The widths of the amide peaks (components 1–3) were constrained to be identical for each spectrum, and the widths of the ester peaks (components 4–5) were likewise constrained. The inferred trans:cis ratio (component 1:component 2) is indicated in the figure.

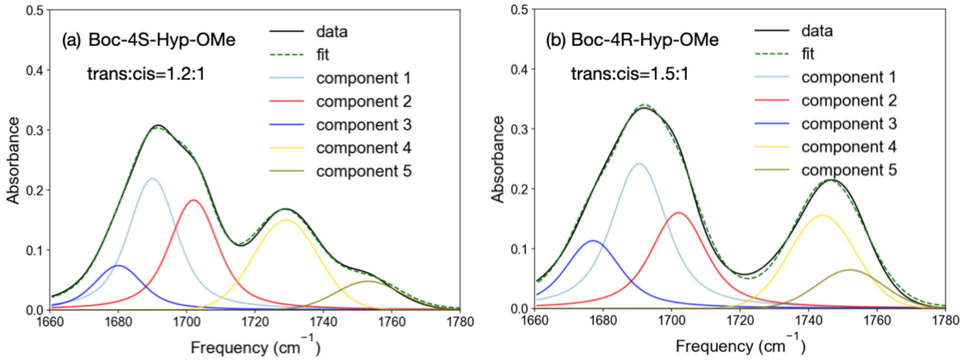

Supplementary Figure 2 The result of the peak fitting of (a)Boc-4R- and (b)Boc-4S-Hyp-OMe at 100 mM concentration using Voigt line shapes. Each spectrum of Boc-4R- and Boc-4S-Hyp-OMe was fitted individually using Voigt lineshapes. The peak positions were fixed based on the location of the minima in the second derivative spectra. The widths of the amide peaks (components 1–3) were constrained to be identical for each spectrum, and the widths of the ester peaks (components 4–5) were likewise constrained. The inferred trans:cis ratio (component 1:component 2) is indicated in the figure.

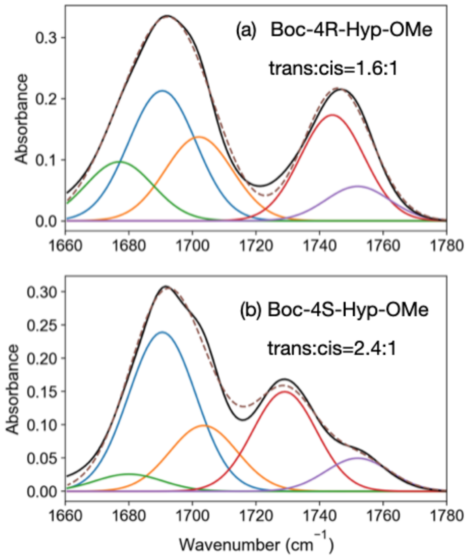

Supplementary Figure 3 The result of the global peak fitting of (a)Boc-4R- and (b)Boc-4S-Hyp-OMe at 100 mM concentration using Gaussian line shapes. Both spectra of Boc-4R- and Boc-4S-Hyp-OMe were fitted simultaneously using five Gaussian lineshapes. The peak positions were fixed based on the location of the minima in the second derivative spectra. The widths of the amide peaks (components 1–3) were constrained to be identical for both spectra, and the widths of the ester peaks (components 4–5) were likewise constrained. The inferred trans:cis ratio is indicated in the figure.

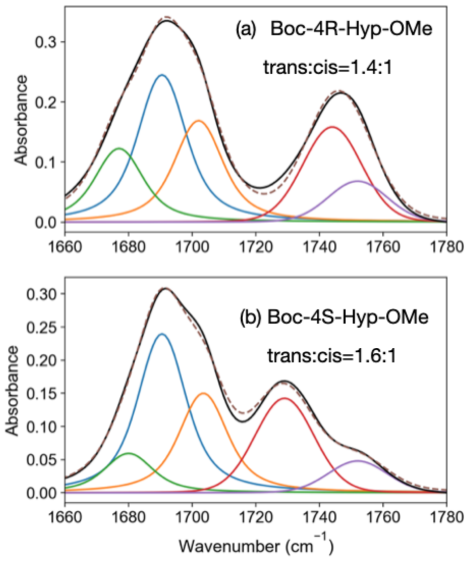

Supplementary Figure 4 The result of the global peak fitting of (a)Boc-4R- and (b)Boc-4S-Hyp-OMe at 100 mM concentration using Voigt line shapes. Both spectra of Boc-4R- and Boc-4S-Hyp-OMe were fitted simultaneously using five Voigt lineshapes. The peak positions were fixed based on the location of the minima in the second derivative spectra. The widths of the amide peaks (components 1–3) were constrained to be identical for both spectra, and the widths of the ester peaks (components 4–5) were likewise constrained. The inferred trans:cis ratio is indicated in the figure.

Supplemental Note 4: 2D-IR spectra in parallel polarization

We present the 2D-IR spectra of Boc-4S- and Boc-4R-Hyp-OMe in parallel polarization, which were used to calculate the anisotropies shown in Figures 3 and 4 in the main text.

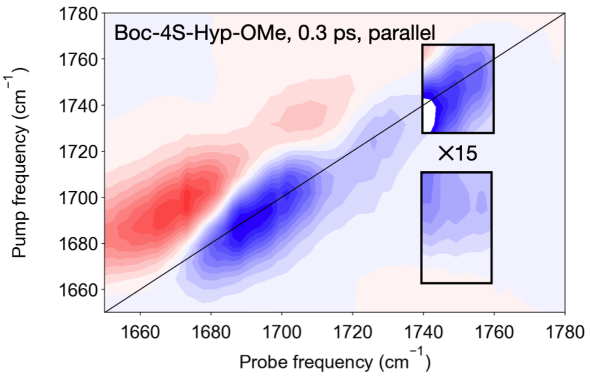

Supplementary Figure 5 2D-IR spectra of Boc-4S-Hyp-OMe in chloroform at 200 mM concentration in parallel polarization at a waiting time of 0.3 ps.

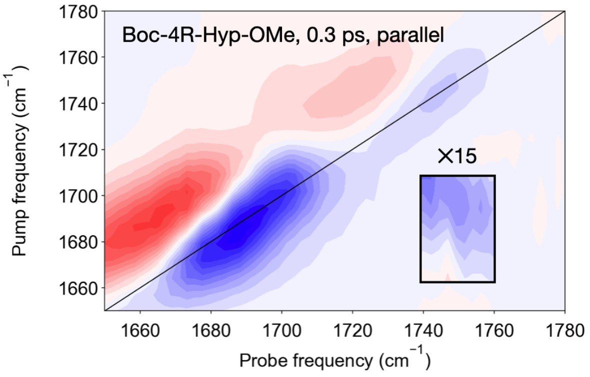

Supplementary Figure 6 2D-IR spectra of Boc-4R-Hyp-OMe in chloroform at 200 mM concentration in parallel polarization at a waiting time of 0.3 ps.

Supplementary references

[1] Neugebauer, J., Reiher, M., Kind, C. & Hess, B. A. Quantum chemical calculation of vibrational spectra of large molecules—raman and ir spectra for buckminsterfullerene. *J. Comput. Chem.* **23**, 895–910 (2002).
