# Supplementary material for: Stereoelectronic and hydrogen-bonding effects on hydroxyproline conformation
Source: Commun Chem. 2026 Mar 24;9:179. doi: 10.1038/s42004-026-01984-x (PMC13184006; doi:10.1038/s42004-026-01984-x)
Supplement: Supplementary file 3 — Description of Additional Supplementary Files [file 42004_2026_1984_MOESM3_ESM.pdf]

Description of Additional Supplementary Files:

File: Supplementary Data 1

Description: structures obtained from our DFT calculations

File: Supplementary Data 2

Description: source data used to plot figures
